# Implementation of a Biomedical Research Training Program for Early-Stage Undergraduate Students to Inspire Development of a STEM Professional Identity

**DOI:** 10.1007/s43683-026-00219-8

**Published:** 2026-03-19

**Authors:** Melissa Richards, Kwadwo Appiah-Kubi, Jennifer Ball, Ali Boolani, Melissa King, Sarah Treptow, Laurel Kuxhaus, Bethany Almeida

**Affiliations:** 1 Institute for STEM Education, Clarkson University, Potsdam, NY 13699 USA; 2 Department of Physical Therapy, Clarkson University, Potsdam, NY 13699 USA; 3 Department of Arts, Culture, and Technology, Clarkson University, Potsdam, NY 13699 USA; 4 Human Performance and Nutrition Research Institute, Oklahoma State University, Stillwater, OK 74078 USA; 5 Department of Chemistry and Biochemistry, Clarkson University, Potsdam, NY 13699 USA; 6 The Clarkson School, Clarkson University, Potsdam, NY 13699 USA; 7 Office of Strategic Coordination, National Institutes of Health, Bethesda, MD 20892 USA; 8 Department of Chemical and Biomolecular Engineering, Clarkson University, Potsdam, NY 13699 USA

**Keywords:** Training program, Undergraduate research, STEM identity, Community of practice, Asset-based mentoring, Cognitive apprenticeship theory

## Abstract

**Purpose:**

This study sought to develop an undergraduate research training program for early-stage students with interest in biomedical engineering. The ultimate goal was to foster a sense of self-identity within the participants as STEM professionals, develop their professional and technical skills, and provide training in biomedical engineering research to under-resourced student populations that would not otherwise be exposed to biomedical engineering research until late in their careers.

**Methods:**

The training program was intentionally designed to leverage and synergistically combine the following pedagogical frameworks: Cognitive Theory Apprenticeship, Asset-based Mentoring, and Community of Practice. The program was designed as a two-year program starting in the pre-first-year summer with a bridge program, research rotations in laboratories throughout the first year, a cohort-led research project in the second year, and a four-course sequence emphasizing the biomedical research process, development as scholars, and networking.

**Results:**

All members of Cohort 1 successfully completed the program, with 2 out of 3 participants transitioning into the university’s Honor’s Program. All members of Cohort 2 successfully completed half of the program prior to early programmatic termination by the funding agency due to changes in federal initiatives. Further, all participants self-reported an increase in self-identity as a STEM professional, with a majority of participants reporting continued interest in research, the biomedical field, and STEM.

**Conclusions:**

This program provided participants from under-resourced backgrounds with the resources and opportunities to succeed, and these students exemplified the potential of participants in similar programs, evidenced by the outstanding research performance achieved by the students.

## Introduction

Preparing the next generation of biomedical engineering professionals is not only vital for the future workforce but is also a national and educational priority. Among all professions, bioengineers and biomedical engineers are expected to see some of the fastest growth rates, at 5% over the next 10 years, with a projection approximating that 1300 jobs will be needed annually due to retirements and occupational transitions [1]. While the USA has historically been a leader in biomedical research [2], sustaining this leadership requires intentional educational structures that foster successful transition into advanced degrees and research careers. These educational structures are necessary avenues to support students entering the bioengineering and biomedical engineering (BME) workforce [3–5].

Literature shows that providing a structured and intentional pathway for undergraduate students into research not only strengthens their STEM identity but also aids in the retention of these students as future STEM professionals [6–10]. Specifically, Thiry et al. (2012) note that undergraduate students new to research who participate in multi-year, sustained research experiences have significantly greater cognitive, personal, and professional gains than their peers with shorter experiences [11]. Furthermore, Haeger & Fresquez (2016) found that the quality and type of mentoring that students receive strongly shapes the knowledge that students gain [12].

However, institutional barriers in the selection of participants to these programs and mentoring processes in helping students identify opportunities have often restricted entry into these research experiences [13–17]. These barriers are especially prevalent for students from underrepresented backgrounds (e.g., first generation, under-resourced schools). Less than 5% of first-year students participate in undergraduate research; among those who do, there is a 10% difference between first-generation students and non-first-generation students [18]. These challenges are particularly common within the bioengineering and biomedical fields, exacerbated by the fact that only 149 of the more than 4000 universities in the USA grant BME undergraduate degrees [19]. For students at non-BME granting institutions, such as Clarkson University, exposure to research in the biomedical field may not happen until either late in their undergraduate studies through external professional opportunities (such as internships or summer research opportunities) or even early in their graduate careers. Therefore, programs that provide students with earlier exposure to research and mentorship than is typical for most undergraduate experiences remains vital. In particular, to achieve lasting impact, such programs must be intentionally designed to ensure equitable access that connects the strengths and lived experiences of all students to research opportunities, but especially those from educationally underserved groups, as they represent a pool of talent that can help address the need for more BME practitioners.

**Fig. 1:**
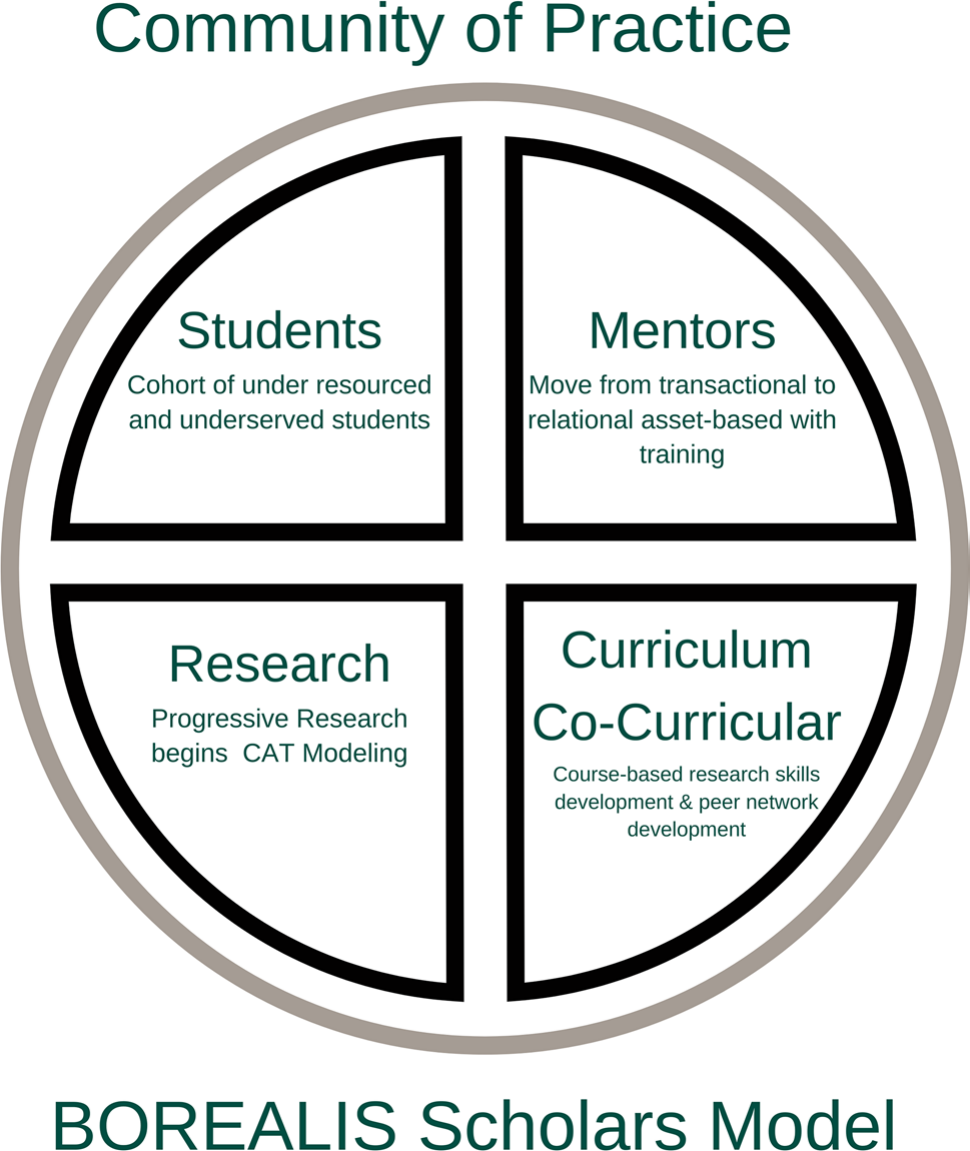
Schematic depicting how Cognitive Apprenticeship Theory (CAT) and asset-based mentoring are leveraged and intertwined within the BOREALIS Scholars Program, a Community of Practice (CoP) Model, to develop scholars into STEM professionals that self-identify as biomedical researchers. The BOREALIS Scholars Program activities are purposefully designed with the CAT model to develop over the first two years and prepare scholars to enter the Honors Program with strong experience and preparation in research. The CoP provides the scholars with a network of like-minded peers and mentors that help them build their skills, and asset-based mentoring focuses on leveraging the scholars’ individual skills and experience through a more personalized mentoring relationship to develop their confidence and competence as biomedical researchers and STEM professionals. The model allows both the scholars and mentors to develop new skills and improve research pathways into biomedical research by allowing quicker entry into research and better supported development of STEM identity by the scholars

The program described in this work, the BiOengineering Research Education to AcceLerate Innovation in STEM (BOREALIS) Scholars Program, was rooted in the Cognitive Apprenticeship Theory (CAT) framework [20, 21], supported by the intentional development of a Community of Practice (CoP) [22, 23] and an asset-based (or strength-based) mentoring model [24–27] (Figure 1). Through the six teaching methods of CAT, students build skills, engage in positive self-reflection, develop critical thinking, foster a STEM identity, and gain agency over their learning [28]. Further, Wenger (1998) notes that CoPs have three defining dimensions: joint enterprise (a shared understanding of the community’s purpose), mutual engagement (common resources and practices that the group develops), and shared repertoire (the collective knowledge and skills that the group possesses) [22]. Leveraging the CAT framework and CoP was a major key to the success of the BOREALIS Scholars Program, allowing scholars and faculty mentors to collaborate and learn from one another by sharing knowledge, skills, and experiences, thereby allowing the scholars to build a stronger STEM identity and navigate the challenges of entering a research-based environment. Finally, the integration of asset-based mentorship reinforces the CoP with a more developed, personal relationship with the students.

Further, the program aimed to address institutional barriers by offering an intentional faculty training program in effective, asset-based mentoring techniques, resulting in the development of a sustainable, inclusive program. The innovation of our program lies in modifying and blending evidence-based strategies to suit the unique environment of a small, rural university and creating a sustainable mentoring education program with lasting benefits. Ultimately, following Bensimon’s organization learning perspective, this type of work is driven by the need to “change the system, not the student” [29]. In this Innovation article, we demonstrate how leveraging these pedagogical frameworks and best practices can be used to develop and implement a successful early-career biomedical research training program. We also provide a discussion of program limitations and recommendations.

## Methods

The BOREALIS Scholars Program at Clarkson University engaged students from the summer before their first year through their rising third-year summer via a Summer Bridge Program, research rotations, and a custom course sequence. Students who were recruited to the program participated in on-campus research during both the summer and the academic year while engaged in a rich curriculum designed to provide exposure to the breadth of contributions that biomedical engineers make to society. This study was approved by Clarkson University Institutional Review Board (Protocol #23-32.2E).

### Scholar Recruitment and Selection

BOREALIS scholars were recruited in the spring prior to their matriculation, with the goal of students having offers to join the program well before “Decision Day.” Working in partnership with Clarkson University’s admissions office, our two-stage interview process began with a survey of applicants who indicated interest in an engineering major to determine if they met the eligibility criteria. Eligibility for the BOREALIS Scholars Program was established to meet the funder's requirements, with the intent to provide a new pathway and mentored undergraduate research experiences to students who might not otherwise have access. The eligibility requirements for applicants included U.S. citizenship, an interest in biomedical engineering, no admittance to the university’s Honors program, and inclusion as a member of a historically underrepresented group (e.g., socio-economic status, disability status, or first-generation college student). All applicants who met these criteria were invited to a virtual mentoring session with the PI and Co-Is. This mentoring session provided an opportunity to discuss best practices in interview preparation while also detailing the BOREALIS Scholars Program expectations and incentives. After this meeting, those who continued to express interest in the program were then interviewed by the PI, Co-Is, a representative of the Honors program, and a representative from the Community of Underrepresented Professional Opportunities (CUPO). Applicants were evaluated based on a rubric emphasizing eligibility (merit and programmatic) and interest in pursuing research activities. The top three applicants were accepted into the BOREALIS Scholars Program; others who were deemed qualified were placed on a waiting list.

In total, the BOREALIS Scholars Program supported two cohorts (*n*=3 per cohort) of scholars. Four scholars identified as female and one scholar self-reported a disability. Furthermore, of the six scholars, three were first-generation college students. Given that Clarkson University does not offer a major degree program in BME and instead offers only a minor, students majored in Mechanical Engineering (3), Aerospace Engineering (1), Civil Engineering (1), and Environmental Engineering (1), and all declared a BME minor. In addition to their major requirements, the BME minor includes two core courses, Introduction to Biomedical Engineering and Biomedical Engineering Fundamentals, Anatomy and Physiology with lab, and 2 BME-related electives, at least one of which must be from an engineering department. The scholars received a stipend, paid monthly, contingent on participation in BOREALIS Scholars Program activities and research rotations.

### Summer Bridge Program

For 5 weeks prior to matriculating at Clarkson, the scholars engaged in an immersive Summer Bridge Program, where they participated in their first research rotation with a faculty mentor as well as took preparatory courses to aid in the high school to college transition. The preparatory course was delivered in conjunction with Clarkson’s long-standing springboard program. Further, given the short duration of the research experience, the first research rotation emphasized replication of prior work, which is aligned with the NIH Director’s current priority of training future biomedical scientists [30]. Students presented their work at Clarkson’s Summer Research and Project Showcase.

### Research Rotations

During their first summer, students were matched with faculty research mentors based on their ranking of interests following a brief presentation about the various faculty research areas. Two additional research rotations in the labs of different faculty research mentors were completed over the course of their first year, with an expected 10 hr/week commitment. In their second year, the students designed and conducted their cohort-led research project. The scholars were also expected to complete one summer research experience (40 hrs/week, 10 weeks), either on campus, in industry, or at another institution in their rising third-year summer. Students were required to present their work, either at Clarkson’s Spring and/or Summer Research and Project Showcase or at a national conference.

### Course Sequence

We created a four-course sequence in BME designed to teach the biomedical research process to scholars, from research study conceptualization to design and experimentation to dissemination. Each course was worth 1 credit, and the collection of courses could be used to fulfill an undesignated elective requirement toward the students’ major degree programs. There were two main student learning objectives for this course sequence: planning, conducting, and disseminating a cohort-led research project and engaging with the broader biomedical engineering community through journal clubs to gain a breadth of knowledge regarding the various types of biomedical research that can be done.

During the first semester, scholars learned to identify, describe, evaluate, and critique different research methods and study designs. They were also introduced to ethical dilemmas faced by biomedical researchers. In this first course, the scholars worked among their cohort to collaboratively identify a research question, develop a hypothesis, and identify a study design to test their hypothesis. During the second course, the scholars were introduced to principles of study processes, including an introduction to descriptive and inferential statistics and measures of central tendency. The scholars also learned about biases in research, such as sampling and estimation bias. For cohort-led projects focused on human subjects, the scholars were also introduced to Institutional Review Board (IRB) documentation, and the scholars worked with their supervising faculty mentor to submit the forms. The third and fourth course emphasized data collection, analysis, and dissemination. During the third course, scholars were given more in-depth information about statistical analysis. Finally, the fourth course entailed completion of data collection and analysis and emphasized dissemination of their findings through a draft journal article manuscript.

In addition to the cohort-led research project, the course sequence also entailed journal clubs where scholars would read a peer-reviewed, original research manuscript. The senior author on the article was invited via Zoom to participate in the journal club activity. In contrast to traditional journal clubs previously described in the literature wherein the invited speaker presents their research [31], in these journal clubs, the scholars presented on the invited speaker’s research article, followed by an open discussion with the invited speaker on their research and their career development and progression. Further, the invited speakers were all external to Clarkson University to build upon the students’ larger community.

### Mentorship

Mentorship was an essential component of the BOREALIS Scholars Program, built into the program at various levels. Prior to mentoring BOREALIS scholars, BOREALIS Program Directors (PI and Co-Is) provided training to the faculty research mentors focused on culturally responsive and asset-based mentoring best practices. This training was designed and conducted in collaboration with the Clarkson University Office of Access Initiatives. There were three sessions, 1 hour each, that engaged the faculty research mentors in dialogue regarding best practices. Faculty research mentors continued to be engaged weekly during ‘coffee hours,’ which formed as informal mentorship sessions with the Program Directors to address concerns or issues, as well as maintain real-time assessments of scholars’ progress in the research rotations. These faculty research mentors then provided direct mentorship to scholars through minimum weekly meetings with the scholars throughout the duration of the research rotation. Prior to starting the research rotation, the faculty research mentor and the scholar completed a mentoring compact. This mentoring compact provided the scholars and their faculty research mentors the opportunity to openly discuss topics such as goals of the research rotation, communication procedures, accommodations, and frameworks for how to handle conflict. The mentoring compact format was adopted from similar documents in other programs to the early-stage undergraduate experience.

In addition to direct mentorship from faculty research mentors, the scholars also received mentorship from the Program Directors. The Program Directors served as the instructors for the course sequence, achieving Program Director-cohort mentorship. In addition, the scholars were paired with a Program Director who was not the instructor for their programmatic course each semester and met with that Program Director one-on-one bi-weekly. This allowed the scholars to interface with various members of the staff and receive feedback both in group settings and individually. The choice to have the individual meetings be with a Program Director who was not their direct instructor was intentional, as well, to allow the scholars freedom to discuss academic concerns as needed. Finally, scholars also received peer–peer mentorship, both within their own cohorts and across cohorts. This peer mentorship was established through the cohort-led research project and interactions between cohorts at journal club activities, social events, and a monthly breakfast check-in event.

### Program Evaluation

**Fig. 2:**
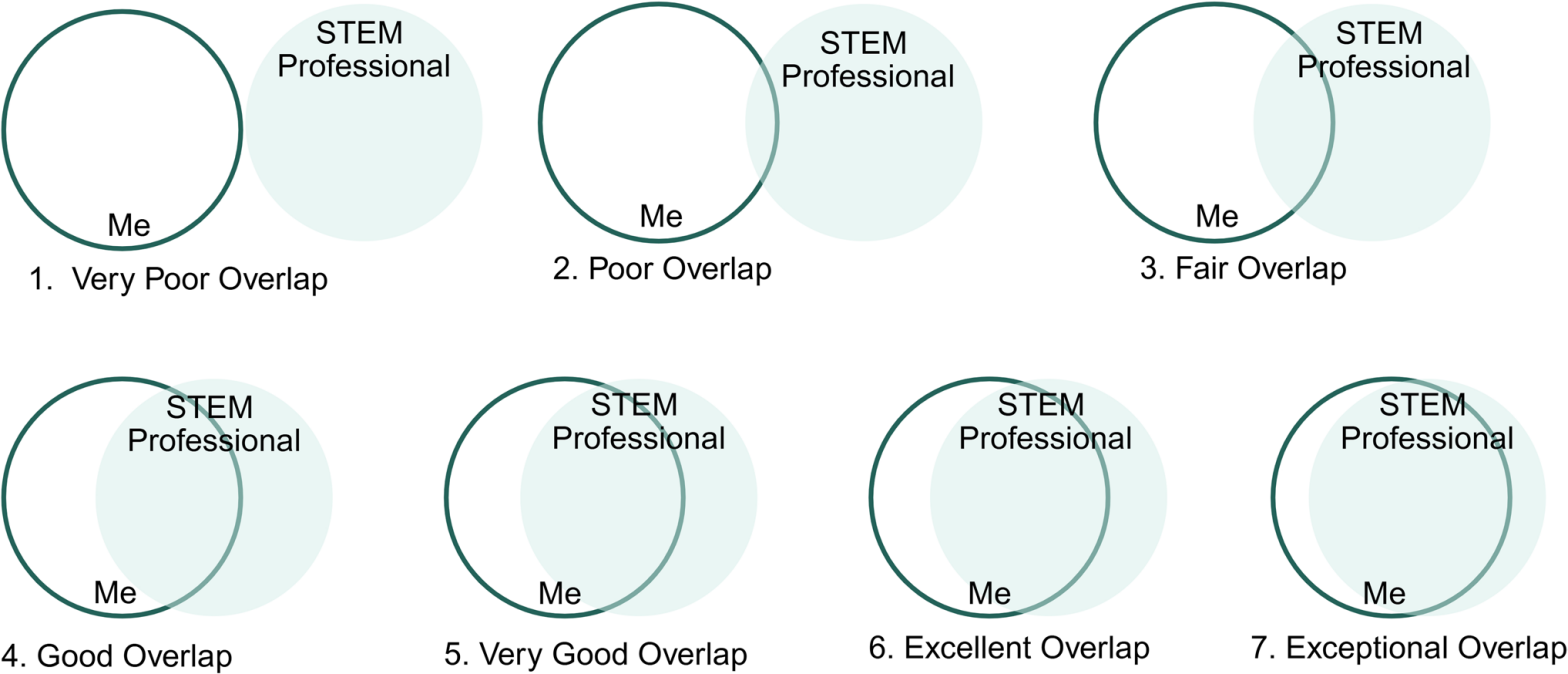
STEM Professional Identity Overlap (STEM-PIO-1) Measure. Scholars selected one of seven pairs of circles labeled “Me” and “STEM Professional,” which varied in their degree of overlap from 1 = Very Poor Overlap to 7 = Exceptional Overlap, to indicate how strongly they perceived their personal identity to overlap with that of a STEM professional (greater overlap = stronger STEM identity). Adapted with permission from the authors, CC BY License, Reference: [32]. Created with Biorender

Upon completion of the Summer Bridge Program, scholars completed a survey designed to determine the effectiveness of the Summer Bridge Program activities. Scholars also completed a second survey upon termination of the BOREALIS Scholars Program (Year 2 for Cohort 1 and Year 1 for Cohort 2) to provide evaluation on their experiences and benefits of the program. A primary outcome of the BOREALIS Scholars Program was developing a sense of STEM identity within the scholars. In both surveys, the scholars were asked to self-identify their perception of self with that of an identity of a STEM professional using the McDonald et al. (2019) single-item STEM Professional Identity Overlap measure (STEM-PIO-1) assessment (Figure 2).

In addition to surveys completed by the scholars, the Higher Ed Insight (HEI) Team, an independent external evaluator, assessed the program through separate surveys and interviews with scholars and faculty mentors. Assessment was broadly based on scholar experience, program implementation, scholar development, faculty mentor perspectives, and recommendations.

## Results

Both cohorts were successfully recruited at the maximum allowable cohort size, 3 students per cohort, as limited by funding. Cohort 1 completed both years of the BOREALIS Scholars Program, while Cohort 2 only completed years 1 out of 2 prior to program termination by the funding agency secondary to EO 14151 [33]. Here, we report on results from both years of the program.

### Summer Bridge Program

**Fig. 3:**
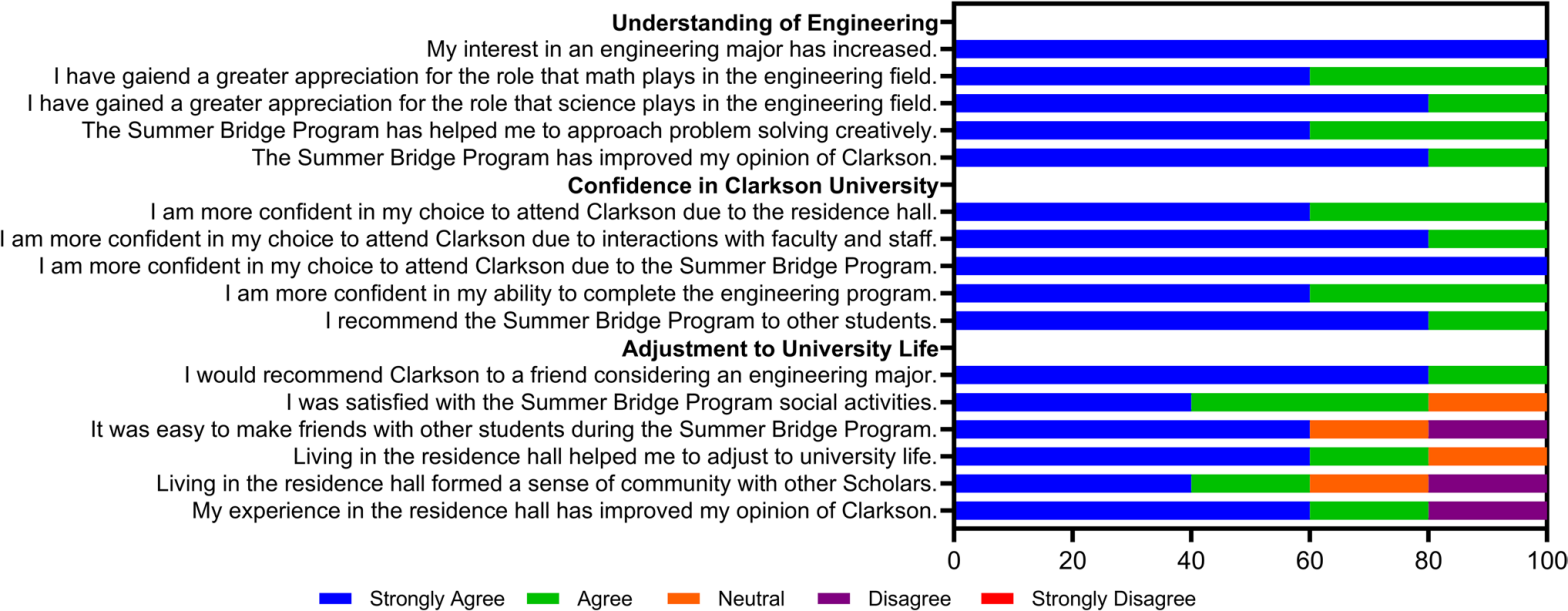
Likert scale evaluating the effectiveness of the Summer Bridge Program. *n*=5 respondents across both cohorts

Five out of six scholars completed the post-Summer Bridge Program survey. Scholars were instructed to rate statements categorized into three thematic topics, an understanding of engineering, confidence in selection of Clarkson University, and adjustment to university life, on a Likert scale from Strongly Agree to Strongly Disagree (Figure 3).

With respect to statements regarding an understanding of engineering, 5 out of 5 scholars indicated that they strongly agreed or agreed with the statement, demonstrating that the Summer Bridge Program was successful in maintaining student interest in engineering and helping the scholars develop a deeper appreciation for the roles of Math, Science, and creative problem-solving in engineering. With respect to statements regarding whether scholars remained confident in their decision to attend Clarkson University, 5 out of 5 scholars again indicated that they strongly agreed or agreed with the statement, demonstrating that the Summer Bridge Program was an effective method to begin to develop the student’s identity as a Clarkson University student, as well as develop the scholars’ sense of cohort.

Conversely, with respect to statements regarding an adjustment to university life and socialization, responses were mixed. All Cohort 1 scholars strongly agreed or agreed with each statement, whereas two Cohort 2 scholars were neutral or disagreed. Of these two, one selected disagreed for at least one item, while the other was never lower than “neutral.” Additional questions within the survey asking scholars what they wish there were more of further demonstrated that 4 out of 5 respondents desired more social events, 5 out of 5 respondents desired more professional events, and 4 out of 5 respondents desired more mentor meetings. Given the small cohort size, the program intentionally collaborated with other campus bridge and enrichment programs (e.g., Honors, McNair, and Springboard) to co-develop weekly professional and social activities and build community. The respondents expressed a desire for program-specific professional, social, and mentoring events focused solely on the BOREALIS cohort identity, rather than shared or co-sponsored programming. Therefore, the feedback reflects a need for dedicated, identity-centered programming.

### Course Sequence

All scholars participated in a 4-course sequence, 1 course per semester, over the duration of the BOREALIS Scholars Program. Cohort 1 completed the entire sequence, and Cohort 2 completed the first two courses when the program’s funding was terminated. These courses, entitled ‘Introduction to Biomedical Research I/II/III/IV’ were designed to introduce scholars to the research process and develop their capacity and network as biomedical researchers. All scholars successfully passed each course.

Cohort 1 successfully planned, conducted, and disseminated a cohort-led research project entitled ‘Effects of ADHD medications on postural balance in young adults’. Cohort 2 successfully planned a cohort-led research project entitled ‘Effects of Music Stimuli on Working Memory and Pupillary Dilation: Investigating the Old vs. New Effect’, developing a research proposal as part of the course sequence. However, Cohort 2 was unable to conduct the research and disseminate their findings due to program termination.

**Fig. 4:**
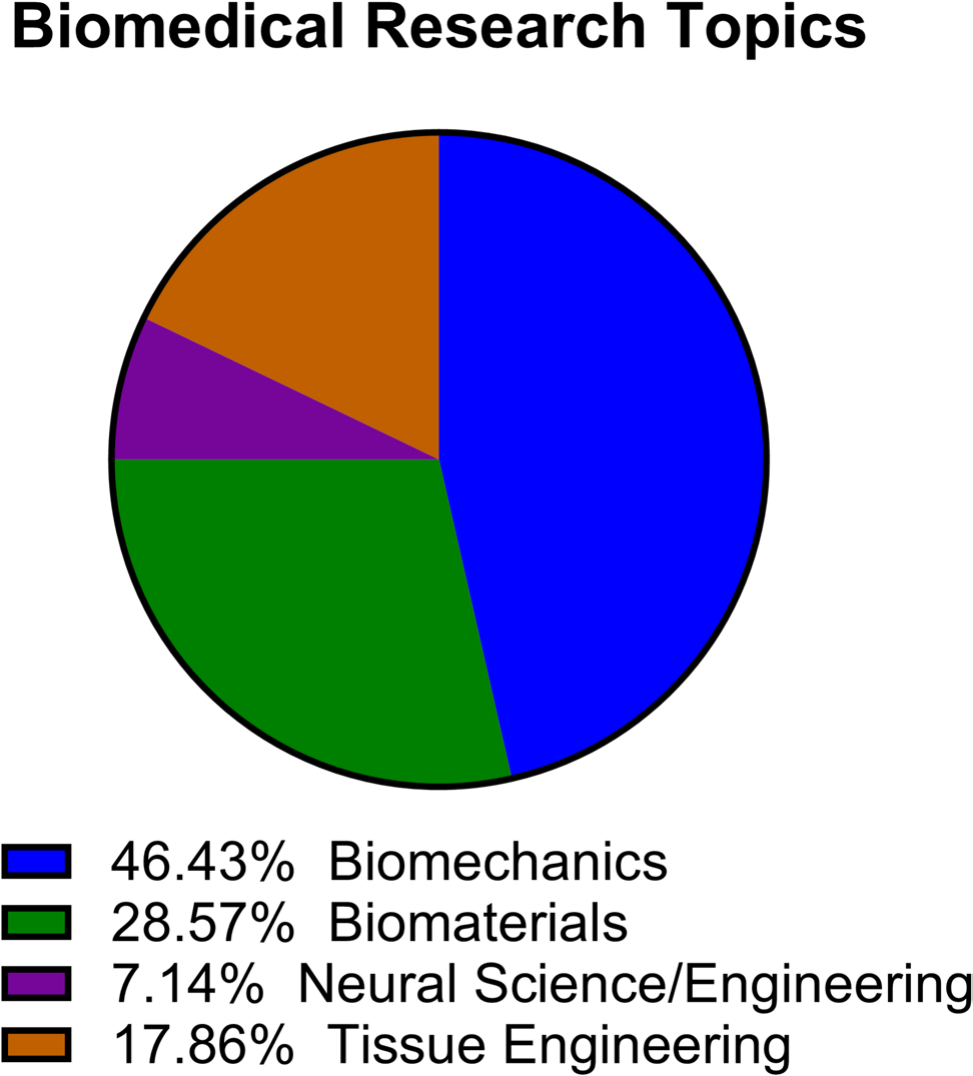
Distribution of biomedical research topics represented in the 21 journal club articles discussed across the four-semester BOREALIS course sequence. Invited speakers often worked across multiple areas, but primary topics were categorized into four themes. This breadth of topics exposed scholars to a diverse range of contemporary biomedical engineering research areas

Over the course of 4 semesters, the scholars engaged in 21 journal clubs. All students successfully completed their journal club presentations each semester with a passing grade. Program faculty recruited the invited speakers by identifying authors of recently published literature in biomedical engineering journals, as well as by leveraging personal networks and existing social media networking platforms (e.g., the BME Women Faculty UNITE Slack channel, NewPI Slack, the BME Women Faculty Facebook Group, and/or #BlackInBiomechanics). The roster was balanced holistically over the course of each semester to include speakers at all career stages, from a diverse array of genders, racial and ethnic backgrounds, academic ranks, and institutions. Of the 21 invitees, 48% identify as male and 52% identify as female. In addition, over 70% of the invited speakers self-reported from groups underrepresented in biomedical engineering. Further, 28% were at the Assistant Professor rank, 24% were at the Associate Professor rank, and 48% were at the Full Professor rank. There were 4 biomedical research topic themes, with some invitees working in multiple themes (Figure 4). Forty-six percent of the research articles were in biomechanics, 29% were in biomaterials, 7% were in neural engineering, and 18% were in tissue engineering and regenerative medicine.

### Research Experiences

All scholars were engaged in semesterly research rotations, in addition to their own cohort-led group project. As mentioned, only Cohort 1 were able to see their group project through to completion prior to funding termination. Cohort 1 conducted a group research project on the effects of ADHD medications on postural balance in young adults. Scholars had experience working with individuals using biophysiological balance equipment such as force plate technology. The scholars disseminated their findings for both the research rotations and their group project in internal and/or external scientific conferences. Notably, at the Clarkson University Research and Projects Showcase (RAPS) annual event, Cohort 1 students presented three times each throughout the BOREALIS Scholars Program, with 2 out of 3 of the scholars receiving a ‘Best Presentation in Session’ award at one of the presentations. Cohort 2 students presented once each at RAPS.

For the external conferences, 2 out of 3 members of Cohort 1 and 1 out of 3 members of Cohort 2 presented at the 2024 National Biomedical Engineering Society (BMES) Meeting and 2 out of 3 members of Cohort 1 presented at the 2024 Northeast Bioengineering Conference (NEBEC). Notably, one scholar contributed to a published, peer-reviewed research article as a result of their work during a research rotation.

### Final Program Evaluation

Five out of six scholars completed the post-Summer Bridge Program survey, and 6 out of 6 scholars completed the final program evaluation survey that contained the single-item STEM Professional Identity Overlap measure (STEM-PIO-1) assessment (Figure 2). After the Summer Bridge Program, 1 out of 5 scholars self-identified as a ‘2,’ whereas a larger proportion of scholars, 4 out of 5 (2 students each), self-identified as a ‘4’ or ‘5,’ giving an average score of ‘4.0 ± 1.2.’ After completion of the program, there was a non-statistically significant increase in the average score to ‘5.3 ± 1.4,’ with 4 out of 6 scholars identifying as a ‘4’ or ‘5’ and 2 out of 6 scholars identifying as a ‘7.’ It is likely that if both Cohorts were evaluated at the end of their full program, the shift would be greater and potentially statistically significant.

When asked to evaluate their plans for the rest of their time at Clarkson, the majority of scholars identified themes regarding their academic interest, persistence in STEM majors, and research engagement (Table 1). All scholars plan to continue in their current STEM majors, with a strong emphasis on biomedical engineering (4/6) and continued engagement in research (3/6). Participation in the honors program (2/3) was also viewed as a valued part of their academic trajectory.

**Table 1:** Thematic summary of the future plans of the BOREALIS scholars

Theme	Number of responses
Continued interest in research	3/6
Interest in biomedical field	4/6
Plan to stay in STEM major	6/6
Honors program involvement	2/3 of those who fully completed the program

**Fig. 5:**
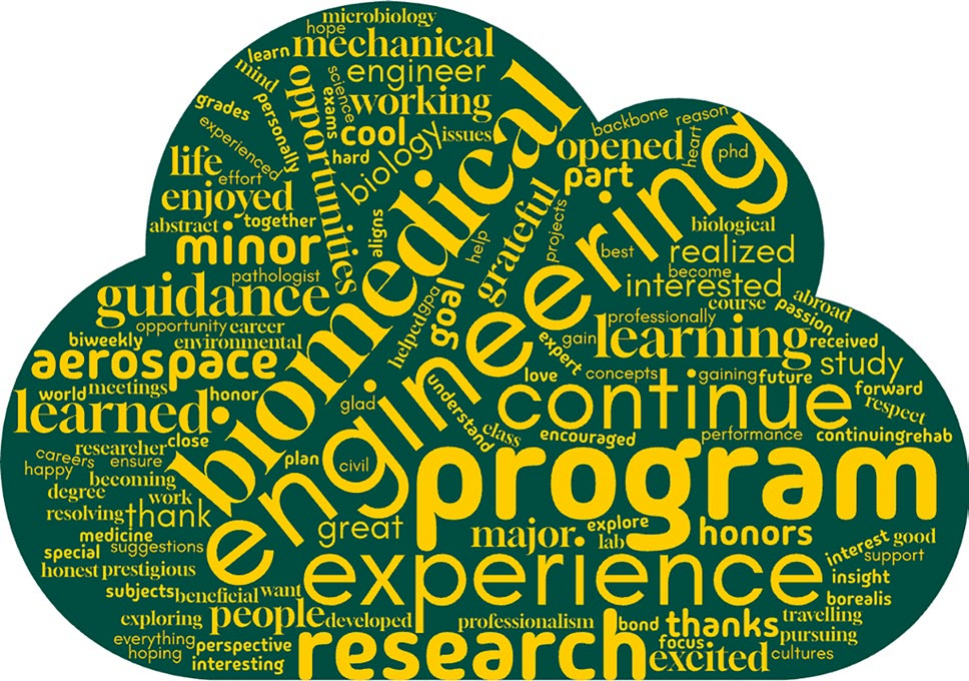
Word cloud demonstrating the most commonly used words and phrases in scholars feedback and suggestions (*n* = 6 respondents across both cohorts). Frequently used terms (e.g., program, engineering, biomedical, research, experience, guidance, learning) highlight overwhelmingly positive perceptions of the program’s value, including learning and research opportunities, mentorship and guidance, personal and academic growth, and motivation toward future STEM and graduate study

As part of the program evaluation, scholars were asked to provide feedback, suggestions or comments that could be used to improve the program. Their responses covered a variety of themes including personal growth and learning, program value and opportunities, gratitude and appreciation to faculty, mentorship and guidance, impact on future goals, program challenges, networking, and connections (Figure 5). All scholars expressed overwhelmingly positive experiences, emphasizing learning, mentorship, and gratitude. They mentioned that the program had given them opportunities to learn a lot and had increased their academic motivation, confidence, and led to clearer career goals. One scholar even expressed interest in pursuing a PhD program in a STEM area (biomedical or aerospace engineering). While scholars expressed minimal programmatic challenges in their responses, they suggested continued improvement in logistics and communication to strengthen the program. Furthermore, sustaining mentorship beyond the program duration was highlighted as desirable. At the end of the program, 2 out of 3 scholars in Cohort 1 moved on to join the Honors program at Clarkson University.

In the second-year report, the HEI reported that scholars deemed the Summer Bridge program to largely have a positive impact on their early programmatic experiences, including developing a sense of connection with their cohort and being more prepared for college transition (2/3, Cohort 2). In addition, Cohort 2 found the academic preparation course beneficial for their first-year success, specifically by helping them understand basic research principles and prepare them for the 40-hour workweek balance between coursework and co-curricular activities. Furthermore, Cohort 1, who had by then participated in research rotations and a summer research experience, noted that the summer research experience improved their research skills, strengthened their interest in bioengineering and STEM, made them feel prepared to engage in future research opportunities, and allowed them to connect with peers and mentors.

When asked what attracted them to the program, scholars (from both cohorts) ranked the research opportunities as one of the most important factors (ranked 1st or 2nd by 5 out of 6 students). Students also shared that the program’s financial support was important (ranked 1st or 2nd by 3 out of 6 students). Career development was the next most common factor, ranking 1st or 2nd by 2 out of 6 students and 3rd or 4th by the remaining 4 out of 6 students. Despite the popularity of the mentorship components of the program, it was ranked as the least important factor most frequently (2 out of 6 ranked it 3rd or 4th, 4 out of 6 ranked it 5th or 6th).

Based on this assessment, the HEI team provided nine recommendations to support overall program success, including (1) increase academic preparation support in the Summer Bridge program, (2) strengthen peer connections through more structured activities, (3) improve communication of program expectations and workload management, (4) expand support for first-year scholars in navigating mentorship, (5) provide additional graduate school preparation resources, (6) maintain focus on enhanced recruitment strategies to reach rural students and students with disabilities, (7) refine program design to balance academic rigor with scholar well-being, and access outcomes, (8) sustain and highlight the effectiveness of the research rotations/experiences and Journal Club, and (9) maintain strong faculty and program staff mentorship.

## Discussion

What makes programs like the BOREALIS Scholars Program unique is that it creates an earlier, structured pathway for successful transition into and completion of the Honors Program, positioning the students to pursue graduate school in bioengineering or a related field. The BOREALIS Scholars Program provided an opportunity to establish a unique community of practice for first and second-year undergraduate students and create an undergraduate pathway earlier in their academic journey. This can lead to more students engaging in this type of research and being prepared to enter these fields. This new pathway also leveraged existing resources for student success. The research education plan included new initiatives for student success during the first undergraduate years, and faculty training in and use of inclusive pedagogy and effective mentoring.

### Cognitive Apprenticeship Theory

The design of the BOREALIS Scholars Program naturally follows the CAT framework, as discussed in the Introduction. To reintroduce this pedagogical framework, CAT emphasizes student learning of the process by which an expert logically moves from problem to solution rather than simply teaching the solution or the process to get to the solution. In other words, it is a pedagogical framework that emphasizes making critical thinking visible. This is a particularly effective pedagogical framework when teaching something as conceptual and lacking in defined structure and steps as conducting, analyzing, and disseminating novel research findings. This is because CAT takes into consideration the tacit processes that are usually unclearly defined or part of the hidden curriculum by allowing students to observe and model experts.

The six stages of the CAT pedagogical framework include Modeling, Coaching, Scaffolding, Articulation, Reflection, and Exploration. All of the components of the Clarkson University BOREALIS Scholars Program can be aligned with one or more of these stages (Table 2). Here, we break down further how each of these stages are accomplished for each individual aspect of the BOREALIS Scholars Program.

**Table 2:** Alignment of BOREALIS Scholars Program to Cognitive Apprenticeship Theory

Stage	Definition	BOREALIS Scholars Program activity
1. Modeling	An expert explicitly demonstrates the task to the student in order to allow that student to develop a conceptual model of the task	Summer Bridge Program; Faculty–Student Mentorship during Research Rotations; Course Sequence
2. Coaching	The students try their hand at the process and are observed by the experts	Faculty–Student Mentorship during Research Rotations; Course Sequence
3. Scaffolding	Specifically designed activities or group work are utilized to help students better understand the process	Course Sequence; Cohort-Led Research Project
4. Articulation	Dissemination focused on enhancing science communication	Course Sequence; Conference Presentation
5. Reflection	The students self-evaluate their problem-solving process	Course Sequence; Final Program Evaluation Survey
6. Exploration	Students are given freedom to problem solve on their own	Course Sequence; Cohort-Led Research Project

The BOREALIS Scholars Program begins with the Summer Bridge Program. The Summer Bridge Program was designed to help the scholars begin to develop camaraderie among their cohort. Through their very first research rotation, before they even have a chance to step foot in the classroom, the scholars have the opportunity to engage in the first two stages of the CAT by working one-on-one with their research faculty mentor to learn how research is conducted and start to develop the technical skills associated with conducting research. They also have a brief experience with the fourth stage of the theory by presenting their work at Clarkson’s Summer Research and Project Showcase. Based on scholar responses (Figure 3), it is clear that the Modeling and Coaching stages were successful in helping the scholars become more excited about their future path in STEM research, achieving the primary objective of the Summer Bridge Program.

Once the scholars arrived on campus, they began to engage in the remaining programmatic activities. The first activity is the course sequence. In the first course, scholars are introduced to the concept of biomedical research, having had a small taste of it after their Summer Bridge Program. In this course, the scholars learn about the various types of research methodologies, biomedical ethics, and how to develop an idea for a research project. The main objective of the first course is to begin to develop a sense of self-identity within the scholars as a biomedical researcher. In this way, the first course of the sequence was specifically designed with the third stage of the CAT in mind, where the course emphasizes demystifying the biomedical research process. The second course in the sequence begins to utilize concepts from the first two stages of the CAT, Modeling and Coaching, through activities designed to show the scholars what a research proposal is and how to develop one from the idea that they generated in the first course. The third course in the sequence, starting in the scholars second year of the program, continues using themes from the second stage, Coaching, but also includes the third, fifth, and sixth stages, Scaffolding, Reflection, and Exploration. In this case, Scaffolding is not used to demystify the concept and process of biomedical research but instead the activities were purposefully designed with the concept of Coaching in order to lead the scholars from proposal planning to experimentation. In conducting the experiments that they designed, the scholars were then able to engage in Reflection and Exploration. Finally, the fourth and final course of the sequence continued to emphasize themes of Reflection and Exploration but also introduced Articulation in the form of disseminating their novel findings in the form of a manuscript draft and conference presentations. This course sequence was successful in helping the scholars to develop not only their technical and professional skills but also in developing their confidence and identity as biomedical researchers (Figure 2), evidenced by the significant number of local and national scientific conference presentations and awards as well as successful completion of Cohort 1’s manuscript draft.

A separate component of the Course Sequence was the journal club, which primarily focused on the Scaffolding and Articulation stages of the CAT. By interacting with experts in biomedical research from across the nation, the scholars were not only able to build their network, but they were also able to observe the plethora of approaches to biomedical research that exist (Figure 4). The success of this activity is evidenced by the HEI’s recommendation to sustain the effectiveness of the journal clubs, highlighting that the journal club was positively seen as a useful professional development activity by the scholars.

Finally, the last component of the BOREALIS Scholars Program were the research rotations. Scholars completed an on-campus research rotation during the Summer Bridge Program, and both semesters of their first year. Scholars also completed a research experience in the Summer between first and second years, either at Clarkson or elsewhere. These research experiences clearly utilize aspects of the first, second, fourth, and sixth stages of the CAT. The faculty-student mentorship, as well as peer-to-peer mentorship from other students in the research group are effective means of Modeling and Coaching the biomedical research process. The scholars were also given the opportunity for Exploration by working on individually led projects during these research experiences that they then disseminated (Articulation) at various scientific conferences and symposia. The success of the research experiences is evidenced by the number of local and national conference presentations and research awards earned by these scholars doing research as first- and second-year undergraduate students. One scholar even contributed research that led to a second author publication in a peer-reviewed journal.

The ultimate success of the BOREALIS Scholars Program, designed to leverage the CAT pedagogical framework, was further evidenced by the increase in scholar self-identity as a biomedical researcher (Figure 2) and their future plans and reflection of the program (Table 1, Figure 5).

### Asset-Based Mentoring Approach

The BOREALIS Scholars Program also followed an asset-based mentoring approach, which is a mentoring style focused on student strengths, valuable skills, cultural identities, and aspirations rather than perceived deficits and shortcomings [34]. This is a particularly effective mentoring approach in achieving one of the primary goals of the BOREALIS Scholars Program, developing an identity of a biomedical researcher and STEM intellectual within the scholars. Faculty-scholar engagements in individual and group research experiences, programmatic check-ins with the Program Directors, and an emphasized reflection on goal settings, progress, and affirmation during the course sequence and research experiences were designed with an asset-based mentoring approach in mind. Further, peer-to-peer mentoring reinforced identity formation, with scholars supporting each other through the course sequence and through collaborating in the cohort-led research project. The findings of the program underscore the importance of this approach. The surveys demonstrated measurable though not statistically significant improvement in STEM identity scores (Fig 2), with more scholars identifying themselves strongly as STEM professionals at the end of the program compared to entry during the Summer Bridge program. Of note, while Cohort 1 had experienced the full BOREALIS Scholars Program, Cohort 2 was only half-way. It is anticipated that had both Cohorts completed the full program, the results would have been greater and perhaps statistically significant. The final program survey also highlighted increased motivation, confidence, and clarity in future careers, including one scholar expressing interest in a doctoral training in biomedical or aerospace engineering (Table 1, Fig 5). These findings align with literature that shows that STEM identity is a critical predictor of persistence in scientific domains, particularly for students historically underrepresented in engineering [35, 36].

In addition, research rotations and cohort-led research projects provided meaningful opportunities to apply scientific knowledge in interdisciplinary environments. Scholars also demonstrated increased motivation and confidence through dissemination of their findings at internal and external conferences. Such authentic scholarly contributions in the scholars’ first and second years of undergraduate study likely reinforce both competence and identify as STEM professionals. The independent evaluation provided by the HEI team corroborated these outcomes, particularly the importance of the Summer Bridge Program and mentoring processes. By capitalizing on the asset-based mentoring approach, scholars expressed much gratitude and appreciation to faculty, mentorship and guidance, and network and connections with mentors and peers (Figure 5).

### Community of Practice Model

Finally, the BOREALIS Scholars Program was also designed to follow the CoP model, where pedagogy is established through shared participation, peer collaboration, and an ongoing process of engagement with interdisciplinary fields. By partnering with the Clarkson University Honors, Higher Education Opportunity Program (HEOP), and CUPO programs, scholars were exposed to different levels of communities with four concentric circles of relationships that interacted with each other. The smallest, innermost circle (representing the most intimate, designated as 1–5 people) existed in the form of the scholar cohorts, where each cohort interacted within and between each other during the Summer Bridge program, course sequence, and cohort-led research project. Here, the scholars built trust, identity, and learned collaboration, which are critical for developing a culture of persistence in STEM. The next circle (kin/family, 15-50 members) adds to the community the other cohort, program staff, faculty research mentors, and laboratory members met during research rotations. In this circle, faculty-scholar mentoring through teaching, research rotations, and bi-weekly check-ins reinforced community belonging by emphasizing scholar’s strengths and helping to meet set goals [37]. The third circle (village, up to 150 members) is met by extending the cohort network to include journal club invited speakers (course sequence), as well as the internal and external advisory committee members. This layer situated scholars within the intellectual discourse of a STEM professional, enabling them to present research findings of external authors from other Universities and obtain broader perspectives of other faculty in the area of biomedical research. The fourth and largest circle (tribe, >150 members) represents the broader biomedical STEM community. Scholars engaged with their tribe through participation in internal and national conferences (e.g. RAPS, BMES, NEBEC) through dissemination of their research findings. This provided them with the development of professional engagement and discourse with different audiences at different levels of their careers, and creating broader networks that would expose them to future career opportunities. The purposeful utilization of a CoP model was deemed successful in the final program survey (Table 1, Figure 5), where scholars expressed gratitude and appreciation for the networking opportunities that they were presented with. The independent evaluation by HEI, where scholars reported career development as an important aspect of the program and where the HEI specifically recommended to maintain and sustain the journal club and program mentorship activities.

### Study Limitations

There are several study limitations that must be considered. First, we acknowledge a small sample size. Three students per cohort is both a positive and a negative for similar programs. For example, due to the mentoring- intensive nature of this program, a larger cohort of students would have limited the ability to effectively implement the asset-based mentorship model. However, with only 6 students total and the fact that not all students completed every survey, there may be a selection bias in the type of individuals and how those individuals felt about the program. Another limitation was the fact that the grant was terminated in year 2, which did not allow the researchers to longitudinally follow both cohorts to completion of the program. However, the termination of the grant was beyond the control of the PIs.

### Recommendations for Future Programs

In this section, we describe our recommendations for any faculty or staff seeking to implement a similar program at their institution. First, we start with general recommendations. Future programs should aim to include a larger and more diverse sample of students to increase generalizability and develop strategies to improve student participation in program evaluation. We recommend that researchers developing similar programs maintain the mixed methods assessment approach, wherein quantitative surveys are conducted in conjunction with qualitative methods such as interviews of students by parties unaffiliated with the program and/or focus groups with each cohort. Other recommendations include identifying reasons for non-completion of assessment to determine whether missing data may bias results.

Next, we present specific recommendations with respect to the research rotations and faculty mentor feedback. The faculty mentorship component included required weekly meetings and a signed mentorship agreement. This agreement was helpful to facilitate conversation between mentors and mentees at the first meeting with critical components, such as a mechanism by which one or both parties could remove themselves from the current rotation or pursue mediated conflict resolution. Continuing to include a formal mechanism for conflict resolution in a program like this was thought to be necessary and important for the scholars. There was feedback on other components in the rotational mentorship agreement that were suggested to be less valuable during the scholars rotations, such as manuscript publication, which could be removed and then replaced when the scholar has chosen a final research lab. Moreover, graduate students and senior undergraduate researchers were involved regularly in some rotations, teaching laboratory techniques and procedures. This less formal but no less significant role in many of the rotations provided a near-peer mentorship component. This was not formally included but it was suggested it should be included in the rotational mentorship contract. In recognition of the fact that a limitation of our program was the small size, we recommend that future programs consider piloting and assessing scalable mentorship models that retain quality while accommodating larger groups.

Next, we present specific recommendations with respect to the four-course series. Within the grant model, research mentors could request up to $500 per student per rotation for research related expenses. Instructors were not paid separately to teach the courses, as they all served as investigators on the grant, with 0.5 - 1.3 months of salary support as part of their overall grant responsibilities. These responsibilities included course development and instruction, cohort mentoring, and program implementation. The courses themselves, however, are not inherently expensive to offer. The primary costs are associated with mentor time, research consumables, and compliance infrastructure, rather than instructional delivery. Instructional components can be implemented using existing curricular structures with minimal cost. Cohort 2 did not complete the sequence because the courses were developed and offered under the NIH funding mechanism, and when funding was terminated, all grant-related activities were required to cease. Importantly, the courses themselves are not grant dependent in design and can be adopted and embedded within existing curricula (e.g., as research foundations courses or structured seminars), thereby preventing overload and substantially reducing staffing and supply costs. The key takeaway for educators from this paper is that structured, intentional course-based research models and early cohort mentoring can positively impact students’ research identity, confidence, sense of belonging, and research readiness without requiring high-cost infrastructure. The outcomes that matter can be achieved through scalable, curriculum-embedded models, with mentored lab research and external funding functioning as enhancements to delivery rather than prerequisites for implementation.

Next, we present specific recommendations with respect to modifications to program structure in order to increase professional development opportunities, as many scholars indicated a desire for more. The program as structured contained two primary avenues for professional development activities: the Journal Clubs, which was noted as a positive experience for scholars by HEI, and discussing professional development topics during the first-year course sequence (e.g., biomedical ethics, self-care, and best practices in teamwork and collaboration). More professional development activities could be included during the Summer Bridge Program as workshops. In addition, although the Journal Clubs were a positive for scholars, there were 21 over the course of the 4 semesters. The total number of Journal Clubs could be reduced, and some of that time could be re-allocated to other professional development activities. Suggested activities include skill-building activities including technical skills (e.g., coding, data analysis, lab safety), scientific communication (i.e., oral, poster, and written), and career preparation (e.g., resume writing, interview practice, career panels), and emotional intelligence (e.g., self-care and stress management, conflict resolution, etc.).

Finally, we present specific recommendations with respect to contingency planning in the event of cuts to funding or a loss in program support. In an ideal world, we would recommend securing sustained funding and institutional support prior to program implementation to allow for long-term follow-up of all cohorts; institutional support specifically would minimize disruption in the program in case of unexpected funding changes. For example, we had institutional commitment to continue the research stipends of scholars after ‘graduation’ from the BOREALIS Scholars Program during their Junior and Senior Years assuming that they matriculated to the Honors Program and conducted research. This was intended to prevent attrition due to low-income scholars needing jobs while studying and as a way to ensure that students are able to remain involved in research activities. While some of the programmatic things might be difficult to institute depending on funding constraints, the mentorship components of this program could be included at no cost to the institution. Adding a formal mentorship component for all undergraduate students participating in research could be taken from this study and applied universally with minor revisions pertaining to the specific program or university. Facilitating discourse between faculty and students to build a mentor/mentee relationship regardless of their major provides a support system while building the student’s self-confidence and independence both of which are necessary to their success and degree completion [38]. This type of continuous mentorship allows students like those in the BOREALIS Scholars Program to realize their full potential and have greater opportunities upon completion of their degrees.

## Conclusion

The BOREALIS Scholars Program was the first program at Clarkson University to provide an early undergraduate pathway to research activity. Before the inception of the BOREALIS Scholars Program: 1) there were no preparatory paths specifically for BME graduate study (despite growing interest from students); 2) no system existed to identify students interested in such a career path (unless they self-declared the BME minor) and provide them with the coaching, mentoring, and support necessary to ensure entry in graduate school; and 3) there were no programs to identify and engage non-Honors students in research before joining the Honors Program. Ultimately, the BOREALIS Scholars Program was successful, despite its early termination.

The BOREALIS Scholars Program was intentionally designed to follow three primary pedagogical frameworks, Cognitive Apprenticeship Theory, asset-based mentoring, and Community of Practice. Together, these three pedagogical frameworks helped to develop 6 bright young minds into aspiring STEM professionals with the grit, motivation, and confidence to continue down a path of biomedical research. At program termination, all members of Cohort 1 successfully completed the entire BOREALIS Scholars Program, with 2 out of 3 members of Cohort 1 moving to the Honors program starting in the fall of their Junior year. In addition, at program termination, all members of Cohort 2 completed their first year of the BOREALIS Scholars Program. This program provided these students from traditionally underrepresented backgrounds with the resources and opportunities to succeed, and these scholars exemplified the potential of similar programs, evidenced by the outstanding research performance (presentations, awards, and publications) achieved by the scholars in only their first and second years as undergraduate students.

## Data Availability

The data that support the findings of this study are available from the corresponding author upon reasonable request.
